# Soil bacteria are more sensitive than fungi in response to nitrogen and phosphorus enrichment

**DOI:** 10.3389/fmicb.2022.999385

**Published:** 2022-09-23

**Authors:** Youchao Chen, Shuwei Yin, Yun Shao, Kerong Zhang

**Affiliations:** ^1^Key Laboratory of Aquatic Botany and Watershed Ecology, Wuhan Botanical Garden, Chinese Academy of Sciences, Wuhan, China; ^2^College of Life Sciences, Henan Normal University, Xinxiang, China

**Keywords:** nutrient enrichments, microbial diversity, microbial composition, antagonistic effect, riparian ecosystem

## Abstract

Anthropogenic activities have dramatically increased nitrogen (N) and phosphorous (P) enrichments in terrestrial ecosystems. However, it is still unclear on how bacterial and fungal communities would respond to the simultaneously increased N and P enrichment. In this study, we used a field experiment to simulate N and P input, and examined the effects of N and P additions on the abundance, alpha-diversity, and community composition of soil bacteria and fungi in a riparian forest. Six nutrient-addition treatments, including low N (30 kg N ha^–1^ year^–1^), high N (150 kg N ha ^–1^ year^–1^), low P (30 kg P_2_O_5_ ha^–1^ year^–1^), high P (150 kg P_2_O_5_ ha ^–1^ year^–1^), low N+P, high N+P, and a control (CK) treatment were set up. We found that the N and P additions significantly affected bacterial abundance, community composition, but not the alpha diversity. Specifically, 16S, *nirK*, and *nirS* gene copy numbers were significantly reduced after N and P additions, which were correlated with decreases in soil pH and NO^-^_3_-N, respectively; Co-additions of N and P showed significantly antagonistic interactions on bacterial gene copies; Nutrient additions significantly increased the relative abundance of *Proteobacteria* while reduced the relative abundance of *Chloroflexi*. Mantel’s test showed that the alteration in bacterial composition was associated with the changes in soil pH and NO^-^_3_-N. The nutrient additions did not show significant effects on fungal gene copy numbers, alpha diversity, and community composition, which could be due to non-significant alterations in soil C/N and total P concentration. In conclusion, our results suggest that soil bacteria are more sensitive than fungi in response to N and P enrichment; the alterations in soil pH and NO^-^_3_-N explain the effects of N and P enrichment on bacterial communities, respectively; and the co-addition of N and P reduces the negative effects of these two nutrients addition in alone. These findings improve our understanding of microbial response to N and P addition, especially in the context of simultaneous enrichment of anthropogenic nutrient inputs.

## Introduction

Anthropogenic activities, such as the fossil fuel combustion and fertilizer application, have caused substantial nitrogen (N) and phosphorus (P) enrichment in terrestrial and aquatic ecosystems (Li et al., 2016; Liu et al., 2021). The excessive input of N and P may greatly influence plant productivity and biodiversity, and cause serious environmental problems like soil acidification and water eutrophication (Conley et al., 2009; Chen et al., 2015; Storkey et al., 2015). Our understanding on how ecosystems respond to the increased N and P enrichment has mainly been limited to aboveground plant communities and soil biogeochemical cycles (Lu et al., 2011; Simkin et al., 2016; Jiang et al., 2019). Soil microorganisms, dominated by bacteria and fungi, play an essential role in ecosystem functioning, with being key drivers of soil carbon (C) and nutrient cycling and plant growth (Widdig et al., 2020). Therefore, the influence of N and P enrichment on soil microbial communities may determine various ecosystem processes in the ongoing global change. However, despite recent efforts (e.g., Ma et al., 2019; Yu et al., 2021), a comprehensive understanding on how N and P enrichments interactively affect soil microbial communities is still needed in terrestrial ecosystems.

Considerable works have been conducted to explore how N additions influence soil microbial communities (Lu et al., 2021; Yan et al., 2021). These studies generally suggested that N enrichment could affect soil microbial diversity and composition by altering soil pH, plant diversity, and soil N availability (Weand et al., 2010; Yang et al., 2020). For example, a meta-analysis by Wang C. et al. (2018) found that the N enrichment reduced both soil microbial α-diversity and the relative abundance of *Actinobacteria* and *Nitrospirae* across different ecosystems. However, the direction of the influence varied greatly among individual studies, with both negative (e.g., Wang H. et al., 2018) and positive effects (e.g., Zhang C. et al., 2019) have been frequently reported on bacterial and/or fungal diversity. Compared with N enrichment, much less information could be found on how P enrichment affects soil microbial communities. The P additions are expected to influence soil microbial diversity and composition by increasing soil P availability, especially in P-limited ecosystems (Liu et al., 2012). However, some studies also indicated that P enrichment did not influence soil microbial communities because of its non-significant effect on soil pH (Wang H. et al., 2018. These inconsistent results suggest more studies with different ecosystem types and experimental methods (e.g., application rate and experiment duration) are still needed.

Notably, many ecosystems are experiencing simultaneously enrichment of N and P, and quantifying the interactive effects of N and P have been considered to be crucial for predicting the responses of terrestrial ecosystems to nutrient enrichments (Jiang et al., 2019; Yan et al., 2021). Yue et al. (2017) have demonstrated that N+P additions largely caused additive effects on soil C storage at the global scale; and another meta-analysis showed that co-additions of N and P have uniformly synergistic effects on aboveground plant biomass across different terrestrial ecosystems (Jiang et al., 2019). Although several studies reported that P addition had marginal effects on soil microbial composition under N enrichment (He et al., 2016; Wang H. et al., 2018), the interactions of N and P on soil microbial communities have been poorly addressed.

Moreover, microbial groups usually show complex relationships in nutrient acquisition and utilization, it is unlikely that bacteria and fungi respond similarly to N and/or P addition (Pii et al., 2015; Ling et al., 2017). For example, Li et al. (2019) reported that the N addition reduced the soil bacterial richness because of soil acidification but had no effect on fungal biomass. However, a recent meta-analysis showed that soil fungal diversity was more sensitive to N addition than bacterial diversity (Yang et al., 2020). This is supported by Yan et al. (2021), which reported that the N addition showed non-significant effects on soil bacterial richness and community composition but significantly increased soil fungal richness in a temperate meadow. These varying results also suggest that the responses of different soil microbial groups to nutrient enrichment might be ecosystem specific.

Although there have been some studies investigating the effects of N and P enrichment in various ecosystems like tropical forests (Li et al., 2019), croplands (Zheng et al., 2020), and grasslands (Yan et al., 2021), scarce information is available on how N and P additions interactively affect soil bacterial and fungal community in riparian ecosystems. Riparian ecosystems have been considered as the effective sink for N and P pollutants from agricultural runoff (Lyu et al., 2021). Due to the increased agricultural fertilization and atmospheric deposition in global change, riparian ecosystems have been experiencing great amount of reactive N and P inputs (Hefting et al., 2003). However, experimental data on how soil microbial communities respond to simultaneously enrichment of N and P is extremely lacking for this kind of ecosystem, which may increase the uncertainties in evaluating the influences of global change on terrestrial ecosystems. Here, we conducted a field experiment of N and P addition in a riparian forest in Qinling Mountain, where extensively high inputs of chemical fertilizers have occurred. The simultaneous application of N and P fertilizer has caused significant influences on ecosystem processes not only within the agricultural lands but also the adjacent riparian and aquatic systems. Here, our aims were to (1) understand how N and P additions interactively affect soil microbial abundance, diversity, and composition in this typical riparian forest, and (2) examine the key factors in determining the influences of nutrient additions on microbial communities. We hypothesized that the changes in soil available N and P induced by nutrient enrichment could explain the responses of microbial communities. We are particularly interested in examining whether N and P additions have a similar mechanism in affecting soil microbial communities, and whether different microbial groups show different responses to nutrient additions.

## Materials and methods

### Site description and experiment design

The N and P addition experiments have been conducted since the year 2013 in a riparian forest (33°36’23” N, 107°50’46”E, 1,560 m a.s.l) at the south aspect of Qinling Mountains, China. The average altitude of our study site is 1,560 m. This region characterized with warm temperate climate. The average annual temperature and annual precipitation is 11.8°C and 1,200 mm, respectively. The main soil is identified as sandy loam based on the USDA classification. The vegetation type is broadleaf forest. The dominant trees are *Populus purdomii* and *Betula platyphylla*; the dominant shrub is *Fargesia spathacea*; and the herbs are *Eulaliopsis binate* and *Arthraxon hispidus*.

A detailed description of experimental design can be found in our previous work (Chen et al., 2022). Briefly, six nutrient-addition treatments (four replicates) and a control treatment (six replicates) were set up. Control (CK, without nutrient addition), low N addition (LN, 30 kg N ha^–1^ year^–1^), high N addition (HN, 150 kg N ha ^–1^ year^–1^), low P addition (LP, 30 kg P_2_O_5_ ha^–1^ year^–1^), high P addition (HP, 150 kg P_2_O_5_ ha ^–1^ year^–1^), low N + low P addition (LNP) and high N + high P addition (HNP) were included. All the plots were in the size of 20 m × 20 m, which were arranged randomly with buffer zones of 10 m. The N and P fertilizer was chosen as urea and CaH_4_P_2_O_8_, respectively, and was applicated once a month from April to September every year from the year 2013.

### Sample sampling and physicochemical analysis

The 0–10 cm soils were sampled in August 2018 at the peak of growing season. The litter layer was removed before soil sampling. Three soil samples were taken from each plot using soil corers. All the soil samples were passed through 2 mm meshes to remove the visible roots and rocks. Subsequently, one portion of soil sample was directly subjected to DNA extraction; and the other portion was used for determining soil pH, soil moisture, total C, total N, total P, available P and inorganic N (NO^-^_3_-N and NH+ 4-N) as described in our previous work (Chen et al., 2022).

### Soil DNA extraction and quantification of gene abundance

Soil total genomic DNA was extracted from 0.5 g samples using the E.Z.N.A.^®^ soil DNA Kit (Omega Bio-tek, Norcross, GA, United States) according to manufacturer’s instructions. The quality and quantity of the DNA samples was checked using NanoDrop 2000 UV-vis spectrophotometer (Thermo Scientific, Wilmington, DE, United States). Then the DNA extracts were stored at −80°C before use. The abundance of 16S rRNA, ITS, *nifH*, *nirK* and *nirS* gene were determined using real-time PCR (qPCR).

### High throughput sequencing

The three DNA samples from the same plot were pooled for the high throughput sequencing. Primer set 515F (5’- GTGCCAGCMGCCGCGG-3’) and 806R (5’- GGACTACHVGGGTWTCTAAT-3’) were selected for the PCR amplification of the V4 region of 16S rRNA genes, and primer set ITS1F (5’-CTTGGTCATTTAGAGGAAGTAA-3’) and ITS2R (5’-GCTGCGTTCTTCATCGATGC-3’) were selected to amplify the Internal Transcribed Spacer 1 (ITS1) of fungi. Sequencing was conducted on an Illumina MiSeq PE300 platform (Illumina, San Diego, CA, United States) according to the standard protocols by Majorbio Bio-Pharm Technology Co., Ltd., (Shanghai, China). We clustered all the tags of > 97% identity into operational taxonomic units (OTUs) using UPARSE version 7.1. The raw reads were deposited into the NCBI Sequence Read Archive (SRA) database (Accession N number: PRJNA832899 and PRJNA832880).

### Statistical analysis

All analyses were performed using in R 3.2.2 (R Core Team, 2019). The effect size of nutrient additions on 16S, ITS, *nirS*, *nirK*, and *nifH* gene copies was evaluated as natural logarithm of response ratio (lnRR) following Yue et al. (2017). The interaction between N and P addition was calculated as Hedges’d using the equations in Crain et al. (2008) and our Supporting Information. According to Crain et al. (2008) and Yue et al. (2017), the interaction of N+P addition could be classified into additive and non-additive (i.e., synergistic and antagonistic). Specifically, when the 95% confidence interval (CI) of Hedges’*d* crossed zero, the interaction of N+P was additive; when the 95% CI did not cross zero, a) if the effects of single-additions were either both negative or exhibit opposite directions, the *d*_*I*_ of N+P < 0 was synergistic and > 0 was antagonistic, and b) if single-addition effects were both positive, the *d*_*I*_ > 0 was synergistic and < 0 was antagonistic.

One-way ANOVA followed by Duncan’s *post hoc* test was employed to test the difference in soil properties, gene copies, alpha-diversity (Chao1 and Shannon index) and abundant phyla or class among different nutrient treatments. Linear regressions were conducted to examine the relationships between gene copies, diversity index, and soil properties. The differences in microbial composition among nutrient treatments were visualized by non-metric multidimensional scaling (NMDS) ordinations which were based on the Bray-Curtis dissimilarity matrices. Then, these differences were tested for significance by analysis of similarity (ANOSIM). The Mantel correlation was examined to test the associations between the microbial community structure and soil properties.

## Results

### Effects of N and P additions on gene abundance

**FIGURE 1:**
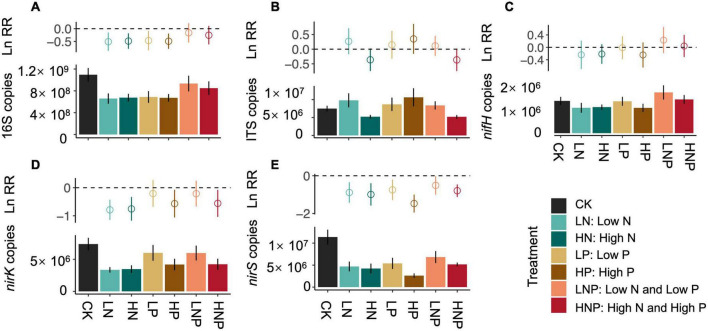
The copy numbers of 16S **(A)**, ITS **(B)**, *nifH* **(C)**, *nirK* **(D)**, and *nirS* **(E)** gene under different nutrient treatments. The effect size of nutrient treatment is showed as natural logarithm of response ratio (lnRR) at the top of each panel.

**FIGURE 2:**
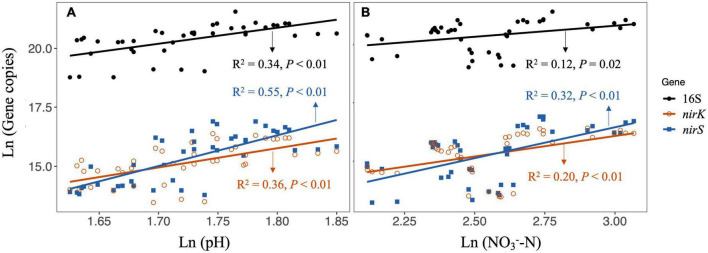
Linear regressions for testing the relationship between soil properties and gene copy numbers of 16S, nirK, and nirS under N additions [**(A)** including CK, LN, and HN treatment] and P additions [**(B)** including CK, LP, and HP treatment]. LN, low N; HN, high N; LP, low phosphorous; HP, high phosphorous; CK, control.

The soil 16S rRNA gene abundance in CK treatment was 1.10 × 10^9^ copies g^–1^ dry soil, and N and P additions in alone significantly reduced the copy numbers (Figure 1A). Significant reductions in *nirK* and *nirS* gene copies after nutrients additions could also be detected (Figures 1D,E)., while *nifH* gene copies were not affected by both N and P additions (Figure 1C). The linear regressions showed that the decreases in 16S, *nirK* and *nirS* gene copies were significantly correlated with soil pH and NO^-^_3_-N under N and P additions, respectively (Figures 2A,B). In contrast to bacterial genes, N and P additions, both in alone and combination, did not affect the copy numbers of ITS gene (Figure 1B). And, the ITS gene copy numbers were negatively correlated with soil C/N across treatments (Supplementary Figure 1).

### Effects of N and P additions on microbial alpha-diversity

For bacteria, the Shannon and Chao1 diversity index ranged from 6.30 (HN) to 6.61 (CK) and 2,620.75 (LN) to 2,779.63 (LNP), respectively; for fungi, the Shannon and Chao1 diversity index ranged from 2.68 (LP) to 4.17 (HNP) and 1,250.52 (LP) to 1,570.60 (HNP). There were no significant differences in alpha-diversity among CK and nutrient addition treatments for both of bacteria and fungi (Table 1).

**TABLE 1:** Alpha diversity of bacteria (16S) and fungi (ITS) under different treatments.

Group	Index	CK	LN	HN	LP	HP	LNP	HNP
Bacteria	Shannon	6.51 (0.03)	6.34 (0.08)	6.30 (0.15)	6.34 (0.21)	6.36 (0.14)	6.50 (0.03)	6.47 (0.03)
Bacteria	Chao1	2,764.71 (35.84)	2,620.75 (102.86)	2,622.9 (139.05)	2,658.61 (159.68)	2,654.47 (151.95)	2,779.63 (55.9)	2,741.27 (86.02)
Fungi	Shannon	3.45 (0.38)	3.23 (0.24)	3.73 (0.24)	2.68 (0.52)	3.56 (0.47)	3.36 (0.28)	4.17 (0.29)
Fungi	Chao1	1,494 (101.24)	1,292.55 (150.82)	1,448.74 (125.4)	1,250.52 (205.36)	1,350.38 (266.23)	1,393.18 (8.15)	1,570.60 (98.04)

Values are shown as mean (standard error). No significant differences in alpha diversity among treatment could be detected (*P* > 0.05) for both of bacteria and fungi. LN, low N; HN, high N; LP, low phosphorous; HP, high phosphorous; LNP, low N and low P; HNP, high N and high P; CK, control.

### Effects of N and P additions on bacterial and fungal compositions

**FIGURE 3:**
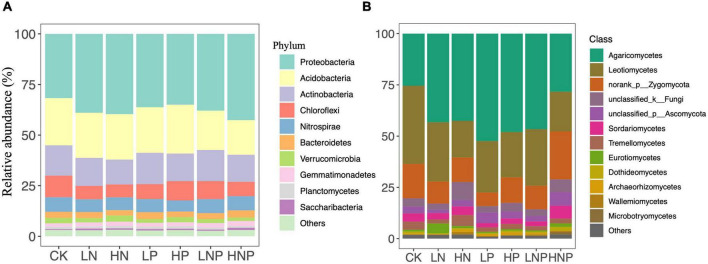
Relative abundances of the soil bacterial **(A)** and fungal **(B)** groups under different nutrient additions. The rare taxa (relative abundances < 1%) are grouped into “other.” LN, low N; HN, high N; LP, low phosphorous; HP, high phosphorous; LNP, low N and low P; HNP, high N and high P; CK, control.

**FIGURE 4:**
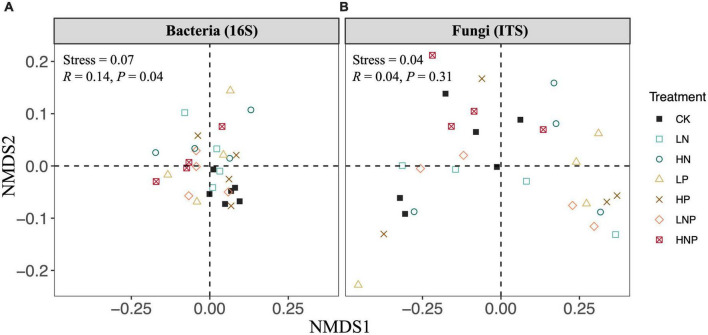
Non-metric multi-dimensional scaling plots (NMDS) for visualizing the differences in soil bacterial **(A)** and fungal **(B)** community compositions across different nutrient additions. The results of analysis of similarity were shown as text in panels. LN, low N; HN, high N; LP, low phosphorous; HP, high phosphorous; LNP, low N and low P; HNP, high N and high P; CK, control.

*Proteobacteria* was the most abundant bacterial phylum across the treatments, accounting for 31.73–42.60% of all taxa, followed by *Acidobacteria* (17.11–23.33%), *Actinobacteria* (12.28–15.39%), *Chloroflexi* (6.35–10.68%), *Nitrospirae* (5.47–7.20%), *Bacteroidetes* (2.61–3.58%) and *Verrucomicrobia* (1.65–3.07%) (Figure 3A). Variations in the bacterial community structures were visualized by NMDS, and ANOSIM suggested that the compositions of soil bacterial communities were significantly affected by nutrient additions (Figure 4A). Specifically, nutrient additions could significantly increase the relative abundance of *Proteobacteria* but reduce the relative abundance of *Chloroflexi* (Supplementary Figure 2).

*Agaricomycetes* showed the highest relative abundance across the treatments, accounting for 25.42–52.41% of all taxa, followed by *Leotiomycetes* (17.88–38.14%), *norank_p_Zygomycota* (6.55–23.39%), *unclassified_k_Fungi* (3.12–8.75%), *unclassified_p_Ascomycota* (1.54–6.52%), *Sordariomycetes* (2.03–6.34%) and *Tremellomycetes* (1.99–5.33%) (Figure 3B). In contrast to the bacterial composition, the fungal composition was not affected nutrient additions (Figure 4B).

**FIGURE 5:**
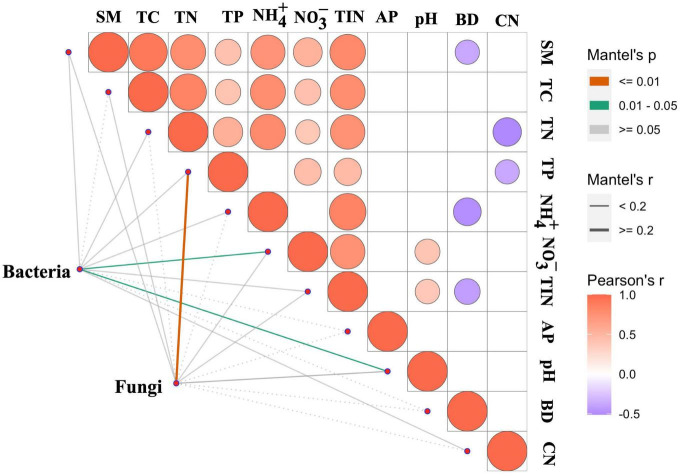
Mantel’s correlation for testing the relationship between soil properties and bacterial and fungal community composition across the nutrient treatments. TC, total carbon; TN, total nitrogen; TP, total phosphorus; BD, bulk density; AP, available phosphorus; SM, soil moisture; TIN, total inorganic N; CN, the ratio of TC:TN.

The Mantel test was employed to examine the relationships between soil properties and soil bacterial and fungal community compositions. The results showed that soil pH (*r* = 0.15, *P* < 0.05) and NO^-^_3_-N (*r* = 0.13, *P* < 0.05) significantly affected bacterial composition (Figure 5), while soil total P (*r* = 0.26, *P* < 0.01) significantly influenced the fungal composition (Figure 5).

### Interactive effects of N and P addition on gene abundance

**FIGURE 6:**
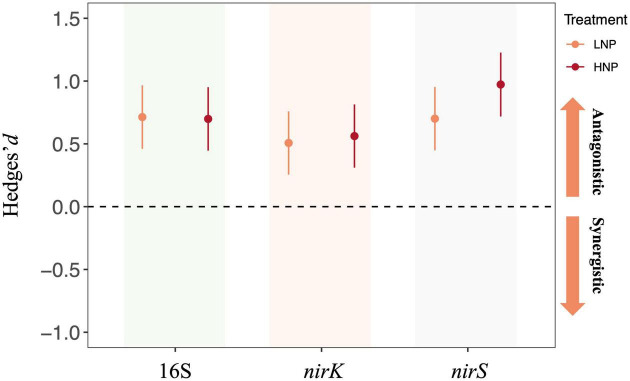
Interactive effects of nitrogen (N) and phosphorus (P) addition on 16S, *nirS*, and *nirK* gene copy numbers. LNP, low N and low P; HNP, high N and high P. Error bars denote 95% confidence intervals.

Our results further indicated that the N and P additions interactively affected the abundance of 16S, *nirK*, and *nirS* gene. Specifically, the Hedges’*d* suggested that the interactive effects of N and P additions were antagonistic for all these three gene copies irrespective of application level (Figure 6).

## Discussion

### N and P enrichment significantly reduced bacterial abundance

The effects of N enrichment on microbial abundance or biomass are confounding in field studies, but significant negative effects have been frequently reported across different ecosystem types (Wang H. et al., 2018; Yang et al., 2021). For example, a meta-analysis by Zhang et al. (2018) showed that the N addition would reduce the bacterial biomass by 16.6% significantly. Here, our results also showed that the N addition in alone reduced the 16S rRNA, *nirS* and *nirK* gene copies (Figures 1A,D,E), which indicated the significant inhibition of N addition on bacterial abundance in this riparian forest ecosystem. The N enrichment could directly or/and indirectly increase microbial C and N substrates that facilitate the growth of microbes, while may also cause soil acidification that inhibits microbial growth (Treseder, 2008). We found that the N additions (i.e., LN and HN) induced significant reductions in soil pH (Supplementary Figure 3); in addition, we also detected significantly positive relationships between soil pH and 16S rRNA, *nirK* and *nirS* gene copies (Figure 2A) across CK and N additions. These results suggested that soil acidification induced by N additions could be the main factor that reduced soil bacterial abundance in this riparian forest ecosystem.

Compared with the microbial response to N addition, the information on how microbes respond to P addition is scarce. Furthermore, the limited experimental data did not reach a consensus on how P addition affects bacterial abundance or biomass. Although soil P availability was increased, Wang Q. et al. (2018) reported that P addition did not influence bacterial abundance, which was supported by some other studies (e.g., Huang et al., 2016). In this study, we also found that the P additions significantly increased soil AP, however, the increased AP did not promote the abundance of bacteria (Supplementary Figure 3 and Figure 1). Interestingly, we found significant decreases in 16S rRNA, *nirK*, and *nirS* gene abundance after P additions, which was correlated with the reductions in NO^-^_3_-N (Figure 2B). The decrease in NO^-^_3_-N after P additions could be due to (1) the inhibition of nitrification as our previous work suggested (Chen et al., 2022), and (2) the stimulated plant N uptake after P limitation was eliminated (Zhang Y. et al., 2019). This result suggested that the mechanism underlying P addition on bacterial abundance could be associated with the reduction of N substrates for bacterial growth, which is quite different from that of N addition as discussed above.

### Antagonistic interactions of N and P on soil bacterial abundance

Terrestrial ecosystem is experiencing the simultaneous alteration of global change drivers, and it is very common to observe the interactive interactions among multiple global change drivers on ecosystem processes (Yue et al., 2017). It has been suggested that synchronously enrichment of N and P has exerted considerable synergistic influence on ecosystems, possibly because the enrichment of a single nutrient leads to the limitation of the alternative nutrient (Elser et al., 2007). This is supported by Jiang et al. (2019), which reported a synergistic effect of co-addition of N and P on aboveground biomass across ecosystems. However, there is a lack of information on how N and P addition interactively affects microbial communities. In the present study, the co-addition of N and P showed antagonistic effects on 16S, *nirK*, and *nirS* gene copies (Figure 6). These results suggested that the negative effects of N or P enrichment in alone on bacterial abundance could be weakened when forest soil receives N and P additions simultaneously. A possible explanation could be that, since soil microbes tend to utilize the nutrients in stoichiometric proportion, the co-addition of N and P could provide bacteria with a balanced supply of nutrients, and this may alleviate the inhibiting effects of N or P addition in alone (Liu et al., 2013). Our findings of the antagonistic effects of N+P addition on soil microbes are particularly important for evaluating global change effects. The above results suggest that the influences of co-addition of N and P on soil microbial communities might not be modeled based on the individual effects of N or P due to their antagonistic interactions. That is, the predicted influences of N+P enrichment on soil microbes could be overestimated if the antagonistic interactions of N and P were not taken into consideration.

### N and P enrichment alters soil bacterial composition but not α-diversity

It has been frequently reported that long-term nutrient fertilization would change soil microbial community composition (Wang H. et al., 2018; Widdig et al., 2020). Similarly, our results showed that N and P additions significantly impacted the soil bacterial composition (Figures 3A, 4A). Particularly, both N and P additions generally increased the relative abundance of *Proteobacteria* while reduce the relative abundance of *Chloroflexi* (Supplementary Figure 2). Our result corroborates previous reports (e.g., Fierer et al., 2012). This finding can be explained by the copiotrophic-oligotrophic hypothesis, in which copiotrophic group like *Proteobacteria* was more likely to grow fast in nutrient-rich conditions, while oligotrophic group like *Chloroflexi* would decline (Fierer et al., 2007; Davis et al., 2011; Li et al., 2019). In addition, changes in microbial composition might be caused not only by the increase of nutrient concentrations in soil but also by the alteration in soil pH (Zhalnina et al., 2015). Our result also showed that pH plays an important role in regulating the bacterial community composition across the different nutrient treatments (Figure 5). Soil pH has been well recognized as a major driver in shaping soil microbial composition at a global scale (Fierer and Jackson, 2006), which could be due to most bacterial taxa exhibiting relatively narrow growth tolerances, especially within the pH range of 4–7 (Rousk et al., 2010).

Although the significant alterations in soil bacterial abundance and composition were detected, our results indicated that neither N nor P additions changed soil bacterial α-diversity significantly in this riparian forest soil (Table 1). This result is inconsistent with some previous studies, in which significant reductions or increases in microbial diversity after N or P additions have been reported (Zhou et al., 2020). Liu et al. (2020) found that soil bacterial diversity did not show significant changes when the N input rates were lower than 160 kg N ha^–1^ yr^–1^, but was substantially reduced because of soil acidification when the N application reached 320 or 640 kg N ha^–1^ yr^–1^. In our study, pH may not be the key factor in influencing diversity, as the N additions induced significant decreases in pH with non-significant effect on bacterial diversity at both 30 and 150 kg N ha^–1^ yr^–1^ application rates. Therefore, we speculated that the controls over diversity may differ from those controls on abundance and community composition. We observed a weak but significant relationship between soil C/N, which was unaffected by nutrient enrichment, and bacterial diversity index (i.e., Chao1 and Shannon index) (Supplementary Figure 4). Similar result has also been reported in Zhalnina et al. (2015). This finding indicated that, although soil C/N has not been recognized as key factor that influence microbial diversity on a continental scale (Li et al., 2019), it may play a significant role in affect bacterial diversity in the riparian forest.

### N and P enrichment did not influence fungal community in riparian forest soil

In contrast to bacterial communities, our results indicated neither N nor P addition significantly affected fungal abundance and community composition (Figures 1B, 3B, 4B), which may suggest that fungal communities generally have higher tolerances to nutrient enrichments than bacterial communities. Previous studies have indicated that the N addition may alter fungal biomass and community composition because of soil acidification (Treseder, 2008). While in our study, fungal abundance and community composition were not affected by the reductions of pH after N addition in this riparian forest ecosystem. A possible reason is that fungal species typically have a wide pH optimum, often covering 5–9 pH units without significant inhibition of their growth (Rousk et al., 2010). Instead, our results showed that soil C/N and TP play a key role in determining fungal abundance and community composition, respectively (Supplementary Figure 1 and Figure 5). The non-significant changes in C/N and TP may explain the marginal influence of nutrient additions on fungal abundance and community composition, respectively (Supplementary Figure 3). In addition, fungal alpha-diversity did not show significant responses to N or P additions, which was similar with bacterial community (Table 1). However, we did not find any relationship between fungal alpha-diversity and the soil properties measured in this study. It has been well documented that fungal diversity is not only regulated by soil physiochemical, but also strongly correlated with vegetation community properties like tree cover, stand density, tree species diversity and composition in forest ecosystem (Tomao et al., 2020). These vegetation community attributes, which remained largely unchanged in our 5-year fertilization experiment, may play more important roles in regulating the responses of fungal diversity to nutrient enrichment. But this speculation needs to be tested in further studies. The above results, corroborates with some other studies (e.g., Li et al., 2019), may suggest that the driving factors of fungal communities could be quite different from those of bacterial communities under nutrient additions.

## Conclusion

This study demonstrated that soil bacteria are more sensitive than fungi in response to N and P enrichment. Specifically, both N and P additions in alone decreased the soil bacterial abundance, which could be due to the reduction in soil pH and NO^-^_3_-N after N and P additions, respectively; The interactions of N and P were antagonistic in affecting the bacterial abundance; Bacterial community composition was also altered by nutrient addition, which was associated with the changes in soil pH and NO^-^_3_-N; The N and P addition did not change the soil fungal abundance, diversity, and community composition. Our results indicated that the enrichment of N and P might influence soil bacteria and fungi through different mechanisms. This work provided new information for understanding the responses of the microbial communities to nutrient enrichment in riparian ecosystem, and highlighted that the interaction in nutrients should be considered in global change studies.

## Data availability statement

The datasets presented in this study can be found in online repositories. The names of the repository/repositories and accession number(s) can be found in the article/Supplementary material.

## Author contributions

YC: writing—original draft, review and editing, formal analysis, investigation, conceptualization, and methodology. SY: writing—review and editing and investigation. YS: writing—review and editing. KZ: writing—review and editing, formal analysis, conceptualization, and funding acquisition. All authors contributed to the article and approved the submitted version.
