# Supplementary material for: Soil bacteria are more sensitive than fungi in response to nitrogen and phosphorus enrichment
Source: Front Microbiol. 2022 Sep 23;13:999385. doi: 10.3389/fmicb.2022.999385 (PMC9537584; doi:10.3389/fmicb.2022.999385)
Supplement: Supplementary file 1 [file Data_Sheet_1.docx]

**Supporting Information**

**Title:**

Soil bacteria are more sensitive than fungi in response to nitrogen and phosphorus enrichment

**Authors:**

Youchao Chen^1^, Shuwei Yin^1,2^, Yun Shao^2^, Kerong Zhang^1,*^

^1^Key Laboratory of Aquatic Botany and Watershed Ecology, Wuhan Botanical Garden, Chinese Academy of Sciences, Wuhan 430074, China

^2^College of Life Sciences, Henan Normal University, Xinxiang, 453007, China

***Corresponding author:**

Kerong Zhang, Email: kerongzhang@wbgcas.cn

*Key Laboratory of Aquatic Botany and Watershed Ecology, Wuhan Botanical Garden,*

*Chinese Academy of Sciences, Wuhan 430074, P. R. China*

*Phone: +86 27 87517163 Fax: +86 27 87510251*

**Emails:**

Youchao Chen ([chenyouchao@wbgcas.cn](mailto:chenyouchao@wbgcas.cn)); Shuwei Yin ([yinshuwei145@163.com](mailto:yinshuwei145@163.com)); Yun Shao (shaoyun73@126.com); Kerong Zhang (kerongzhang@wbgcas.cn)

**Interaction of N and P addition**

The interaction effect size (*d*_I_) between N and P addition was calculated using the following equation:

$$d_{I}=\frac{{(\overline{X}}_{\mathrm{NP}}-\overline{X}_{N})-{(\overline{X}}_{P}-\overline{X}_{\mathrm{Ck}})}{2s}J(m)$$

where $\overline{X}_{\mathrm{Ck}}$ ,$\overline{X}_{N}$, $\overline{X}_{P}$ and $\overline{X}_{\mathrm{NP}}$ represents the mean value of a variable in control, N, P and N+P additions (i.e., LNP and HNP), respectively; *s* is the pooled standard deviation and *J* (*m*) is correction term for small sample bias. The *s and J* (*m*) were calculated using the equations below:

$s=\sqrt{\frac{\left（ n_{ck}-1 \right）s_{ck}^{2}+\left（ n_{N}-1 \right）s_{N}^{2}+\left（ n_{P}-1 \right）s_{B}^{2}+\left（ n_{\mathrm{NP}}-1 \right）s_{\mathrm{NP}}^{2}}{n_{ck}+n_{N}+n_{P}+n_{\mathrm{NP}}-4}}$

$J\left( m \right)=1-\frac{3}{4m-1}$

﻿where *n_ck_*, *n_N_*, *n_P_* and *n_NP_* is the sample size of control, N, P and N+P additions, respectively; *s_C_*, *s_N_*, *s_P_* and *s_NP_* were the standard deviations of the control, N, P and N+P additions, respectively; ﻿*m* was the degree of freedom, which could be calculated as *m* = *n_ck_* + *n_N_* + *n_P_* + *n_NP_* - 4. The variance of $d_{I}$ was calculated according to the equation below:

$v_{2}=\frac{1}{4}\left[ \frac{1}{n_{ck}}+\frac{1}{n_{N}}+\frac{1}{n_{P}}+\frac{1}{n_{\mathrm{NP}}}+\frac{d_{I}^{2}}{2(n_{ck}+n_{N}+n_{P}+n_{\mathrm{NP}})} \right]$

**Figure S1** Relationship between soil C/N and ITS gene copy numbers across different nutrient additions. CK, control; LN, low N; HN, high N; LP, low phosphorus; HP, high phosphorus; LNP, low N and low P; HNP, high N and high P

**Figure S2** Relative abundance of Proteohacteria and Chloroflexi under different nutrient treatments. CK, control; LN, low N; HN, high N; LP, low phosphorus; HP, high phosphorus; LNP, low N and low P; HNP, high N and high P

**Figure S3** Soil properties under different nutrient additions. AP, available phosphorus; TP, total phosphorus

**Figure S4** Lineral regression for testing the relationship between soil C/N and bacterial diversity. CK, control; LN, low N; HN, high N; LP, low phosphorus; HP, high phosphorus; LNP, low N and low P; HNP, high N and high P
